# Exploring optical, electrochemical, thermal, and theoretical aspects of simple carbazole-derived organic dyes

**DOI:** 10.1016/j.heliyon.2024.e25624

**Published:** 2024-02-06

**Authors:** Praveen Naik, Nibedita Swain, R. Naik, Nainamalai Devarajan, Abdel-Basit Al-Odayni, Naaser A.Y. Abduh, Kavya S. Keremane, Devarajan Alagarasan, T. Aravinda, H.B. Shivaprasad

**Affiliations:** a Department of Chemistry, Nitte Meenakshi Institute of Technology, Yelahanka, Bengaluru, 560064, Karnataka, India; b Department of Engineering and Materials Physics, Institute of Chemical Technology-Indian Oil Odisha Campus, Bhubaneswar, 751013, India; c Solid State and Structural Chemistry Unit, Indian Institute of Science, Bangalore, 560012, Karnataka, India; d Department of Restorative Dental Science, College of Dentistry, King Saud University, P. O. Box 60169, Riyadh, 11545, Saudi Arabia; e Department of Chemistry, College of Science, King Saud University, Saudi Arabia; f Materials Research Institute, The Pennsylvania State University, University Park, PA, 16802, USA; g Department of Physics, Nitte Meenakshi Institute of Technology, Yelahanka, Bengaluru, 560064, Karnataka, India

**Keywords:** Carbazole, Computational studies, DSSC, Energy Level Diagram, NLO, Organic dyes

## Abstract

This study highlights the recent advancements in organic electronic materials and their potential for cost-effective optoelectronic devices. The investigation focuses on the molecular design, synthesis, and comprehensive analysis of two organic dyes, aiming to explore their suitability for optoelectronic applications. The dyes are strategically constructed with carbazole as the foundational structure, connecting two electron-withdrawing groups: barbituric acid (**Cz-BA**) and thiobarbituric acid (**Cz-TBA**). These dyes, featuring carbazole as the core and electron-withdrawing groups, demonstrate promising spectral, optical, electrochemical, thermal, and theoretical properties. They show strong potential for diverse optoelectronic applications, promising efficient light absorption and robust stability. The results endorse their suitability for practical optoelectronic systems.

## 1 Introduction

The organic semiconductor materials have attracted researchers across the globe owing to their possible application in optoelectronic devices such as organic light-emitting diodes (OLEDs), organic photovoltaics (OPVs), dye-sensitized solar cells (DSSCs), sensors, and photodetectors etc [[1], [2], [3], [4], [5], [6], [7], [8]]. Organic materials display several advantages such as design versatility, good processability economical device, transparency, and tuneable optical and electrochemical properties, over their inorganic counterpart [[9], [10], [11], [12]]. In recent years, electronic devices employing organic semiconductors have witnessed substantial advancement in terms of the lifetime, stability, and performance of the device [8]. Typically, the sensible molecular design of organic materials employed in the device architecture is the key to achieving ideal optoelectronics properties. A deep understanding of the correlation between the materials molecular structure and their properties is crucial for the development of advanced and efficient devices.

The integration of fused heteroaromatics like indole, carbazole, phenothiazine, and phenoxazine into a “push-pull" architecture has emerged as a highly effective design strategy for creating organic materials with diverse optoelectronic applications [[13], [14], [15], [16], [17], [18], [19], [20]]. Carbazole-based dyes, have garnered significant attention in various optoelectronics contexts, thanks to their remarkable attributes, including facile functionalization at multiple positions, straightforward synthesis, hole-transporting capabilities, and high molar extinction coefficients. These features have rendered carbazole and its derivatives as prominent building blocks for a wide array of optoelectronic devices, capitalizing on their distinctive optical and electrochemical properties [17,[21], [22], [23]]. Notably, the field of nonlinear optical (NLO) materials has also found carbazole-based compounds intriguing due to their distinctive electronic structure and donor-acceptor characteristics [[24], [25], [26], [27], [28]]. By integrating both electron-donating and electron-accepting groups into the carbazole framework, these compounds demonstrate enhanced charge transfer interactions, crucial for achieving efficient non-linear optical (NLO) responses. The electron-rich carbazole unit operates as a donor, facilitating electron transfer to electron-deficient acceptor groups. This molecular architecture not only enhances charge separation but also fosters intramolecular charge delocalization, culminating in superior nonlinear optical properties. As a result, the exploration of donor-acceptor systems built around carbazole holds immense potential for advanced NLO materials, customizable with tailored optical and electronic attributes, positioning them as promising candidates across photonics and optoelectronics [[27], [28], [29], [30][27], [28], [29], [30][27], [28], [29], [30]]. The allure of carbazole and its derivatives further extends to their adaptability and distinctive traits, rendering them appealing for further exploration and development across various domains [[31], [32], [33], [34], [35], [36], [37]]. Current research endeavours concentrate on refining their electronic and optical features, alongside the creation of novel carbazole-based compounds, engineered to deliver enhanced performance and functionality for diverse applications.

The electron-deficient nature of barbituric acid and thiobarbituric acid has garnered significant attention in the realm of optoelectronic devices, due to their exceptional photophysical characteristics [[38], [39], [40]]. Barbituric acid exposed to exhibit high absorbance and emission in the ultraviolet (UV) range. Beyond their applications in solar cells, barbituric acid and thiobarbituric acid have been explored for their potential roles in OLEDs, serving as blue light emitting materials, and as photosensitizers in DSSCs [[41], [42], [43]]. Both barbituric acid and thiobarbituric acid have exhibited good electroluminescence properties, including high brightness and efficiency, and have shown promise as light emitting materials for OLEDs. Overall, the distinctive photophysical and electronic properties of barbituric acid and thiobarbituric acid render them compelling materials for diverse applications in the field of optoelectronics [41,44].

Ongoing research is actively exploring the full potential of these materials in the field, encompassing the development of new derivatives and the optimization of device architectures. In this regard, we report simple carbazole based organic dyes carrying with two withdrawing groups i.e., barbituric acid and thiobarbituric acid. The synthesized dye's structure was validated through spectral analysis. To evaluate their suitability for potential optoelectronic applications, the synthesized dyes underwent thorough examination through photophysical and electrochemical studies. Additionally, the assessment was complemented by Density Functional Theory (DFT) calculations and Time-Dependent Density Functional Theory (TD-DFT) calculations.

## 2 Experimental section

### 2.1 Materials and methods

All materials and solvents were procured from commercial suppliers and utilized without further purification. The molecular structures of **Cz-BA** and **Cz-TBA** were conclusively confirmed using various analytical techniques, including NMR spectroscopy (Bruker 400 and 100 MHz), High-Resolution Mass Spectrometry (HRMS), and elemental analysis (Flash EA1112 CHN analyser). UV–Visible spectra and fluorescence emissions were recorded in tetrahydrofuran (THF) solution at room temperature using a UV–Visible and fluorescence spectrophotometer. Cyclic voltammetry (CV) measurements were performed with a three-electrode setup submerged in an acetonitrile solution containing a supporting electrolyte (0.1 M tetrabutylammonium hexafluorophosphate) at a scan rate of 0.1 V/s, employing a CHI400A electrochemical workstation. To ensure data reproducibility, five complete cycles were recorded. Furthermore, comprehensive DFT and TD-DFT calculations, along with MESP maps, were conducted at the B3LYP/6-31G* level using the BIOVIA Turbomole 2022 software package.

### 2.2 Synthesis

#### 2.2.1 Synthesis of N-ethyl carbazole-6-oxothymine (Cz-BA)

0.25 g (0.001 mmol) of *N*-ethyl carbazole carboxaldehyde was taken in 10 ml of absolute methanol was stirred at 60 °C for 30 min. Subsequently, 0.5 g (0.003 mmol) of barbituric acid was gradually added with continuous stirring. The reaction mixture was then stirred for 10–12 h at 60 °C. Following the reaction's completion, the precipitated solid was filtered and rinsed with pre-cooled ethanol. The obtained dye underwent additional purification through recrystallization in a dichloromethane (DCM)/methanol mixture, yielding a pure yellow solid.

Yield 85 %, ^1^H NMR (400 MHz, DMSO‑*d*_6_, ppm), 11.30 (s 1H), 11.19 (s 1H), 9.30 (d, 1H), 8.65 (dd, 1H), 8.54 (s, 1H), 8.20 (d, 1H), 7.75 (m, 2H), 7.57 (t, 1H), 7.34 (t, 1H), 4.53 (m, 2H), 1.38 (t, 3H). ^13^C NMR (100 MHz, DMSO‑*d*_6_, ppm): 168.3, 164.8, 163.0, 157.4, 150.8, 143.2, 140.8, 133.9, 130.1, 127.3, 124.0, 123.0, 122.8, 121.1, 121.0, 114.6, 110.6, 109.6, 40.1, 40.0, 39.8, 14.3, CHN Analysis analytical calculated for C_19_H_15_N_3_O_3_: C - 68.46; H - 4.54; N - 12.61. found C - 68.43; H - 4.57; N - 12.59. Mass: HRMS 334.1192 [M+1].

#### 2.2.2 Synthesis of N-ethyl carbazole-6-oxo thiothymine (Cz-TBA)

0.25 g (0.001 mmol) of N-ethyl carbazole carboxaldehyde was taken in 10 ml of methanol and stirred at 65 °C for 30 min. Subsequently, 0.5 g (0.003 mmol) of 2-thiobarbituric acid (was added to the clear solution, and stirring was continued for 10–12 h at a constant temperature. The precipitated solid formed was collected, filtered, and washed with pre-cooled ethanol. Subsequently, the obtained residue underwent recrystallization, leading to the formation of a pure red solid.

Yield 92 %, ^1^H NMR (400 MHz, ppm), 12.39 (s, 1H), 12.30 (s, 1H), 9.35 (d, 1H), 8.69 (dd, 1H), 8.55 (s, 1H), 8.21 (d, 1H), 7.76 (m, 2H), 7.56 (t, 1H), 7.36 (t, 1H), 4.53 (m, 2H), 1.38 (t, 3H). ^13^C NMR (100 MHz, DMSO‑*d*_6_, ppm): 192.5, 178.7, 173.3, 163.1, 160.7, 158.5, 143.6, 140.9, 130.7, 128.8, 127.3, 124.2, 123.0, 122.9, 122.8, 121.4, 120.7, 114.6, 110.7, 110.4, 110.1, 109.8, 40.1, 40.0, 39.8, 14.3, 14. 2 CHN Analysis analytical calculated for C_19_H_15_N_3_O_2_S: C - 65.31; H - 4.33; N - 12.03. found C - 65.30; H - 4.36; N - 12.0 Mass: HRMS 350.0963 [M+1].

## 3 Results and discussion

### 3.1 Chemistry

**Scheme 1:**
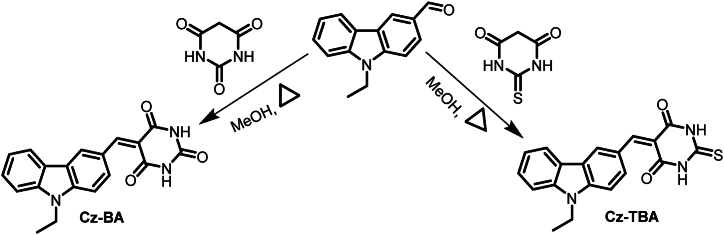
Synthetic routes of the carbazole-based dyes, Cz-BA and Cz-TBA.

The synthesis of **Cz-BA** and **Cz-TBA** dyes followed a straightforward Knoevenagel condensation reaction [45,46], as depicted in Scheme 1. In this synthetic pathway, N-ethyl carbazole carboxaldehyde underwent condensation with barbituric acid and thiobarbituric acid, yielding the targeted **Cz-BA** and **Cz-TBA** dyes, respectively. The synthesis protocol resulted in a substantial yield of the dyes, which were subsequently purified via recrystallization.

#### 3.1.1 Electronic absorption and emission studies

**Fig. 1:**
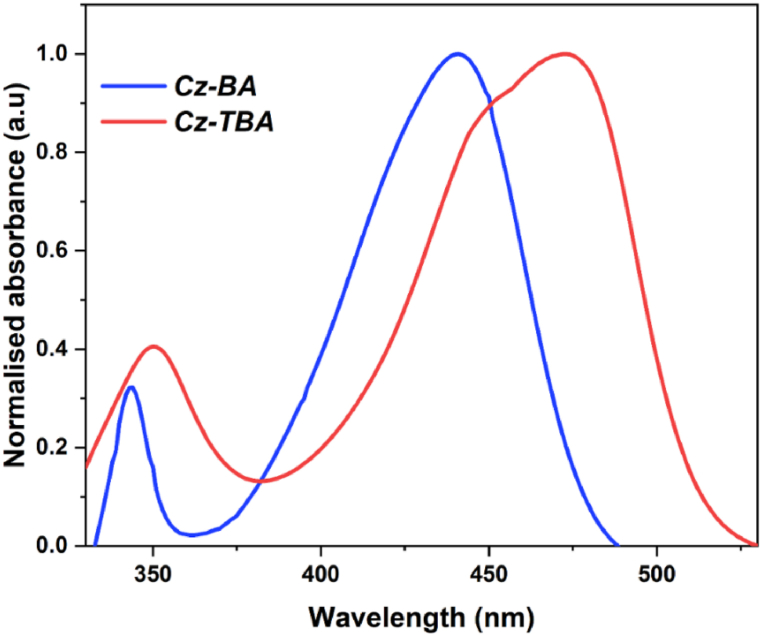
Normalized UV–Visible absorption spectra of **Cz-BA** and **Cz-TBA**.

Fig. 1 depicts the UV–Vis absorption profiles of the **Cz-BA** and **Cz-TBA** dyes, meticulously recorded in THF solvent at room temperature using a Labman UV–Vis absorption spectrophotometer. The pertinent spectral data extracted from these measurements are summarized in Table 1. As illustrated in Fig. 1, both **Cz-BA** and **Cz-TBA** dyes exhibit distinctive dual absorption bands. The absorption peaks observed at 343 nm (**Cz-BA**) and 351 nm (**Cz-TBA**) stem from π-π* electronic transitions originating within the donor component. The absorption signals manifesting at higher wavelengths, specifically at 441 nm (**Cz-BA**) and 473 nm (**Cz-TBA**), can be attributed to ICT (intramolecular charge transfer) phenomena, whereby, electron-deficient barbituric acid and thiobarbituric acid acceptor units engage in interactions with the carbazole donor moiety. Notably, the carbazole dye integrating thiobarbituric acid exhibits a noteworthy bathochromic (red) shift. Furthermore, the optical band gaps (E_0-0_) of **Cz-BA** and **Cz-TBA** dyes were deduced from the normalized absorption spectrum, yielding values of 2.58 and 2.42 eV, individually. Moreover, the molar extinction coefficients (*ε*) for **Cz-BA** and **Cz-TBA** dyes were quantified as 26,543 M^−1^cm^−1^ and 36,490 M^−1^cm^−1^, respectively. These values underscore their remarkable light-absorbing capabilities and signify their potential for efficacious light absorption.

**Table 1:** Optical properties of Cz-BA and Cz-TBA.

Dyes	Cz-BA	Cz-TBA
λ_abs_ (nm)	441	473
λ_em_ (nm)	506	519
*ε*	26,543 M^−1^cm^−1^	36,490 M^−1^cm^−1^
Stoke Shift (nm)	65	46
E_0-0_, Optical (eV)	2.58	2.42

**Table 2:** Electronic parameters obtained from DFT calculations.

Dyes	Bandgap (eV)	HOMO (eV)	LUMO (eV)
Cz-BA	3.23	−5.63	−2.40
Cz-TBA	3.03	−5.68	−2.65

**Fig. 2:**
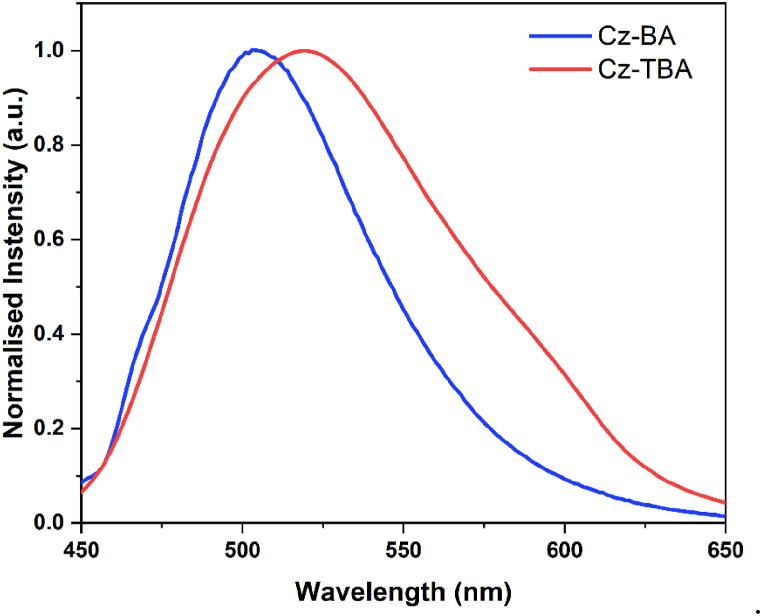
Normalized fluorescence emission spectra of **Cz-BA** and **Cz-TBA**.

Fluorescence emission spectra of Cz-BA and Cz-TBA dyes were precisely recorded in THF solutions, corresponding to their respective excitation wavelengths. Illustrated in Fig. 2, the normalized emission spectra for both Cz-BA and Cz-TBA dyes in THF solution are displayed, with their corresponding spectral values summarized in Table 3. Singular, distinct emission bands were observed at 506 nm (Cz-BA) and 519 nm (Cz-TBA). In addition, their Stokes shift values were calculated using the UV–Vis absorption and fluorescence emission spectra. The computed values were found to be 65 nm for **Cz-BA** and 46 nm for **Cz-TBA**, as detailed in Table 1. The notably larger Stokes shift for **Cz-BA** in comparison to **Cz-TBA** underscores its effective intramolecular charge transfer (ICT) characteristics.

**Table 3:** Electronic absorption parameters obtained from TD-DFT calculations.

Dyes	λ_abs_I (nm)	λ_abs_^S^ (nm)	f = OS^I^	f = OS^S^
Cz-BA	431	431	0.43	0.43
Cz-TBA	461	461	0.51	0.51

^I^ - isolate dye and ^S^ - Chloroform solvent.

### 3.2 Thermal behaviour

**Fig. 3:**
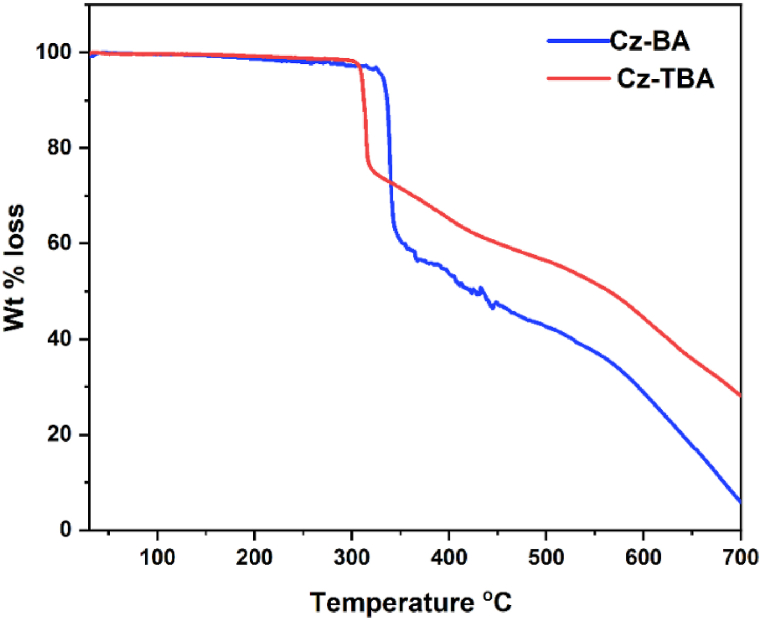
TGA analysis of **Cz-BA** and **Cz-TBA** dyes.

The thermal characteristics and stability of the fluoranthene derivatives were comprehensively examined using TGA analysis. In particular, the decomposition temperature (T_d_) of both **Cz-BA** and **Cz-TBA** dyes were accurately determined through TGA, and the resulting plots are graphically presented in Fig. 3. The experimental procedure involved subjecting the samples of **Cz-BA** and **Cz-TBA** dyes to controlled heating within an inert nitrogen gas atmosphere, with a gradual temperature increase rate of 10 °C per minute. The results indicate that the decomposition temperatures (T_d_ 5 %) corresponding to a 5 % weight loss were found to be 332 °C for **Cz-BA** and 310 °C for **Cz-TBA**, respectively. The notably high T_d_ values exhibited by these dyes hold significant implications, suggesting their suitability for applications requiring elevated temperature operations. This enhanced thermal stability underscores their potential for integration into device fabrication processes that demand resilience in the face of challenging thermal conditions.

### 3.3 Computational studies

**Fig. 4:**
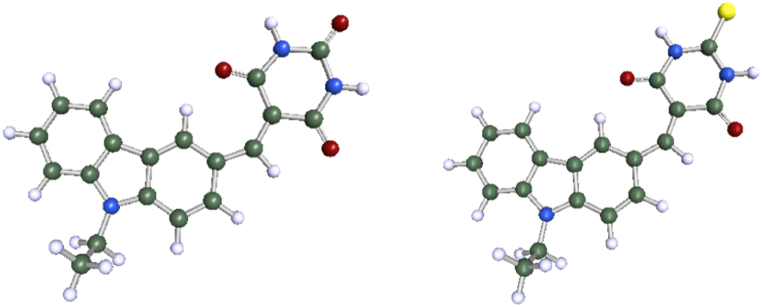
Optimize the structure of the synthesized dyes **Cz-BA** and **Cz-TBA**.

**Fig. 5:**
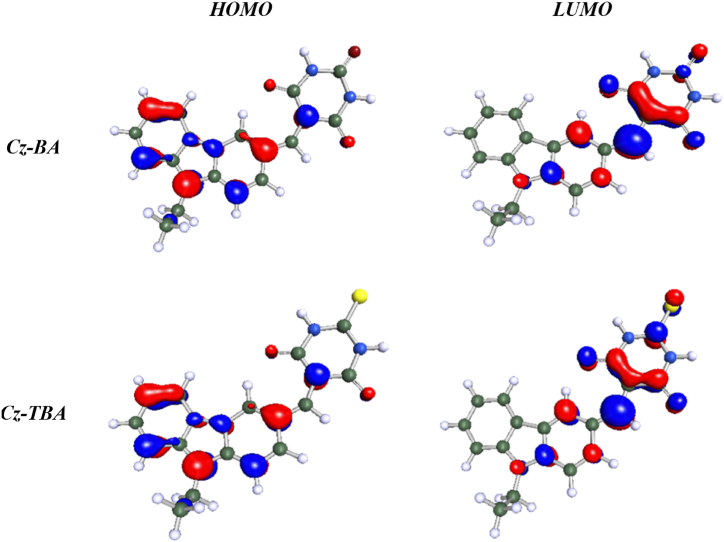
HOMO-LUMO energy levels of **Cz-BA and Cz-TBA**.

DFT calculations were executed to evaluate the electronic feasibility and characteristics of **Cz-BA** and **Cz-TBA** dyes, encompassing both ground and excited states, through the utilization of the Biovia Turbomole 2022 software package [[47], [48], [49]]. Employing the B3LYP/6-31G* level [50; 51], the electron density distribution in the HOMO and LUMO energy levels, along with the optimized geometries obtained from Turbomole, are visually represented in Fig. 4, Fig. 5. Numerical values derived from these calculations are compiled in Table 2. Initially, molecular geometry optimization was conducted at the AM1 semiempirical level. The 3-D optimized structures distinctly portray efficient charge separation in the frontier molecular orbital energy levels. In the HOMO levels, the electron density predominantly localizes on the carbazole units for both dyes, showcasing their electron-rich donor nature. Conversely, in the LUMO levels, the electron clouds notably shift towards the acceptor unit, signifying their effective electron-accepting characteristics. Notably, the calculation underscores that the electron density of the LUMO energy levels in both the dyes has shifted towards the acceptor units.

**Fig. 6:**
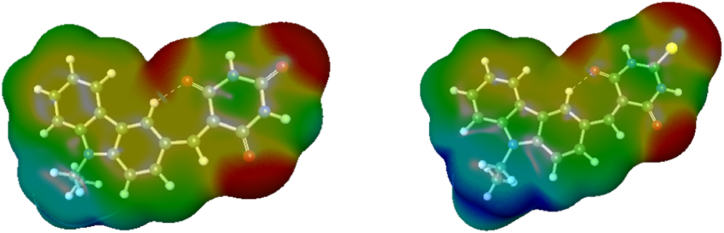
ESP mapping on the iso density surface of Cz**-BA** and **Cz-TBA**.

In Fig. 6, ESP maps of **Cz-BA** and **Cz-TBA** offer a detailed view of the total charge density distribution at various spatial points surrounding the dye molecules. The alignment of cavity boundaries in the molecules with the density isosurface in the ESP plot visually represents the total charge distribution, encompassing electronegativity, dipole moment, and sites of chemical reactivity within the molecule. D**is**tinct colors in the ESP plot correspond to electrostatic potential values, organized in the order of blue > green > yellow > orange > red. Following this color scheme, red and blue contours on the plot denote electron-rich and electron-deficient regions, respectively, indicating the presence of positive and negative charges on the cavity surface. This dynamic interplay establishes local electric fields within the cavity [[52], [53], [54]]. As a result, ESP plots serve as a vivid visualization of the movement of electron density from the donor species to the acceptor/anchoring unit through spacer units. This phenomenon significantly contributes to efficient electron transfer in both **Cz-BA** and **Cz-TBA**, as demonstrated by the distinctive patterns revealed in the ESP maps.

**Fig. 7:**
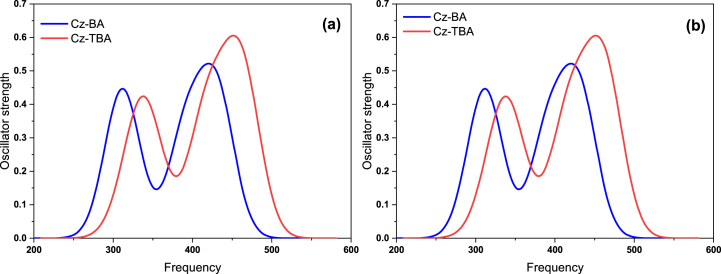
Simulated electronic absorption of Cz-BA and Cz-TBA dyes in (a) gaseous state and (b) THF solvent.

TD-DFT serves as a computational tool for comprehending the electronic behaviours of molecules [[55], [56],], elucidating light absorption in dyes like **Cz-BA** and **Cz-TBA** through absorption wavelengths (λ_abs_) and oscillator strengths (f). This method simulates electronic transitions over time, aiding researchers in optimizing dyes for diverse applications based on their absorption traits. Fig. 7 portrays simulated absorption spectra of **Cz-BA** and **Cz-TBA**, stemming from B3LYP/6-31G* calculations in gaseous and THF solvent phases, detailed in Table 3. Oscillator strength (f) gauges the likelihood of electronic transitions, with higher values indicating higher probability. Remarkably, **Cz-BA** and **Cz-TBA** exhibit identical **λ**_**abs**_ and **f** for isolate (I) and solvent (S) phases, signifying consistent absorption across environments. Fig. 7 further demonstrates two distinct peaks in simulated absorption spectra, correlating with π-π* transitions and ICT phenomena. This observation aligns harmoniously with UV–Vis absorption spectra derived from real-world data, validating the chosen basis set and XC functional. The incorporation of DFT and time-dependent perturbation theory in TD-DFT enables the computation and analysis of absorption properties. By comparing TD-DFT outcomes with experimental data, researchers refine models, gaining enhanced insights into dye electronic structures. This knowledge underpins tasks from developing purpose-specific dyes to tailoring properties for specific technological needs.

### 3.4 Organic electronics applications

#### 3.4.1 Dyes as photosensitizers for DSSCs

**Fig. 8:**
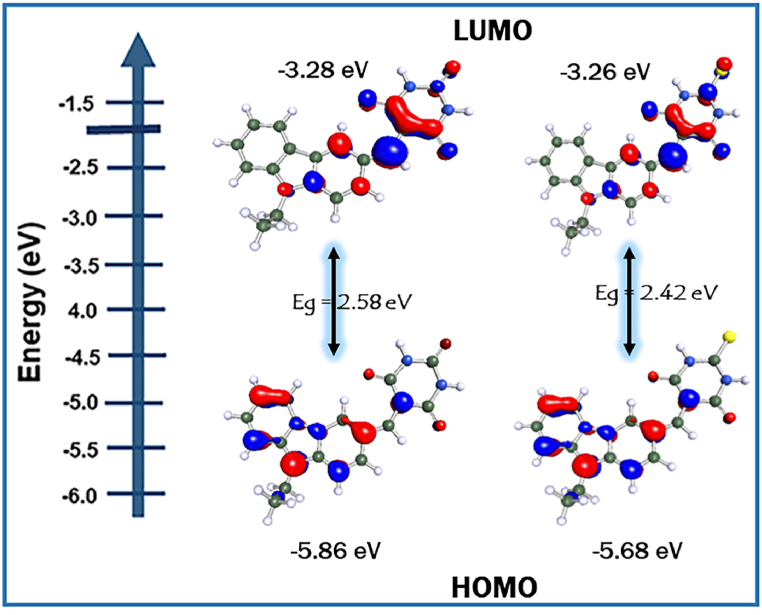
Energy level diagram of **Cz-BA** and **Cz-TBA** dyes.

The energy level diagram illustrated in Fig. 8, along with the comprehensive data provided in Table 4, encapsulates the calculated HOMO-LUMO values and E_0-0_ values for **Cz-BA** and **Cz-TBA** dyes. These values were derived from optical and cyclic voltammogram analyses, with Fig. S7 presenting corresponding cyclic voltammogram traces. This collective information serves as a crucial foundation for assessing the potential suitability of these organic dyes as photosensitizers in DSSC applications.

**Table 4:** Theoretical thermodynamics properties of Cz-BA and Cz-TBA dyes.

Dyes	E^OX^ (V)	HOMO (eV)	LUMO (eV)	ΔG_inj_ (eV)	ΔG_reg_ (eV)	ΔG_rec_ (eV)
Cz-BA	1.23	−5.86	−3.28	0.92	0.66	1.43
Cz-TBA	1.05	−5.68	−3.26	0.94	0.48	1.48

A noteworthy aspect is the positioning of the HOMO energy levels for these dyes, extending deeper than both the Nernst potential of the I₃⁻/I⁻ electrolyte system and the conduction band (CB) of TiO_2_. This characteristic underscores their efficacy in facilitating the regeneration and reduction of the oxidized dye by the electrolyte species. Simultaneously, the estimated LUMO levels are favourably situated relative to the CB edge of TiO_2_, ensuring efficient processes of charge injection and dye regeneration. The oxidation potentials obtained from cyclic voltammetry studies were utilized to calculate their ESOP values using Equation (1).

(1) HOMO = -[E^OX^ - E_1/2_(ferrocene)+ 4.8 eV ].

Here, E^OX^ represents the reduction potential of dyes, and E_1/2_ (ferrocene) is 0.17 eV. Additionally, their GSOP values were computed from the obtained E_0-0_ (optical band-gap) and ESOP values, as expressed in Equation (2):

(2) LUMO = [HOMO - E_0-0_] eV.

To complement this analysis, we conducted calculations of essential thermodynamic parameters, including ΔGreg (Gibbs free energy for dye regeneration), ΔGinj (Gibbs free energy for electron injection), and ΔGrec (Gibbs free energy for charge recombination). Equations (3), (4), (5)) were utilized for these calculations, considering Nernst potentials against the standard calomel electrode (SCE) for the CB edge of TiO2 and the I₃⁻/I⁻ electrolyte system, which are −4.2 eV and −5.2 eV, respectively.

(3) $$\Delta G_{\text{inj}}=E_{LUMO}–\;E_{\text{CB}}\;\left({\text{TiO}}_{2}\right)$$

(4) $$\Delta G_{\text{reg}=}\;E\;\left(I_{3}^{‐}/I^{‐●}\right)\;–{\;E}_{\text{HOMO}}$$

(5) $$\Delta G_{\text{rec}}=E_{\text{CB}}\;\left({\text{TiO}}_{2}\right)\;–{\;E}_{\text{HOMO}}$$

Table 4 summarizes the calculated values of these thermodynamic parameters for **Cz-BA** and **Cz-TBA** dyes, providing a concise overview of their performance in terms of ΔGinj, ΔGreg, and ΔGrec. Significantly, both the dyes exhibit favourable thermodynamic driving forces for charge injection and dye regeneration, meeting the specified range [[57], [58],]. This indicates promising potential for these dyes as sensitizers, as they meet the electrochemical prerequisites necessary for efficient charge transport in DSSC applications.

#### 3.4.2 Refractive index calculation from E_0-0_ through various theoretical model

The optical bandgap and refractive index (n) stand as critical and fundamental parameters in assessing the suitability of any material. Theoretical relationships, particularly equations involving the refractive index and optical bandgap, provide insights into the optical and electronic characteristics of a material. In numerous cases, the refractive index exhibits an inverse relationship with the bandgap. The linear refractive index of **Cz-BA** and **Cz-TBA** can be determined using the well-known Dimitrov and Sakka relation [59].

(6) $$\frac{n^{2}-1}{n^{2}+2}=1-\sqrt{\frac{E_{0-0}}{20}}$$

Here ‘E_0-0’_ represents the optical bandgap. The computed refractive index values are detailed in Table 5. The high-frequency dielectric constant (*ε*_∞_) is determined as n^2^, utilizing the refractive index obtained from equation (1), and the results are provided in Table 5 [60].

**Table 5:** Theoretical calculation of refractive index from models for Cz-BA and Cz-TBA dyes.

Dyes	Refractive index				
	n_[DS]_	n_M_	n_R_	n_[HV]_	n_T_
**Cz-BA**	2.308	2.307	2.007	2.230	2.270
**Cz-TBA**	2.311	2.309	2.013	2.233	2.273

Another approach to establishing a relationship between n and E_0-0_ was undertaken by Moss [60] relying on the material's energy levels. This relationship is expressed as, E_0-0_ x n^4^ = k (constant). Rearranging the above equation,

(7) $$n_{M}=\sqrt[{4}]{\frac{95}{E_{0-0}}}$$

In this equation, the constant k s assigned a value of 95 eV, where n represent the refractive index. This relationship is employed to calculate refractive loss, enhancing the solar cell's conversion parameter. Following this, Ravindra [61] proposed a modification to the aforementioned relation, presenting an alternative model that accounts for the constant difference between the average and optical energy gaps. The relation is expressed as:

(8) $$n_{R}=4.084-\left[0.62\times E_{00}\right]$$

Moss confirmed the validity of this relation for bandgaps less than 4 eV; however, it may generate impractical values for extremely low and high optical energy values. Herve-Vandamme [62], relying on oscillator theory, introduced an alternative relation suitable for materials with low optical energy gaps:

(9) $$n^{2}=1+{\left(\frac{A}{E_{00}+B}\right)}^{2}$$

Here, A is 13.6 eV, representing the ionization energy of hydrogen. The relation can be expressed as:

(10) $$n_{\left[HV\right]}=\sqrt{1+{\left(\frac{13.6}{E_{00}+3.47}\right)}^{2}}$$

In addition to the aforementioned relations, Tripathy [63] proposed an exponentially decreasing relationship between n and E_00_ that characterizes the refractive index of materials in relation to their corresponding bandgaps:

(11) $$n_{T\;}=1.73\times \left[1+1.9017\times e^{-0.539\times E_{00}}\right]$$

The theoretical values of ‘n' are presented in Table 5. It is evident that the calculated ‘n' values for **Cz-TBA** are higher than those for the **Cz-BA** material. This observation aligns with the inverse relationship between ‘n' and E_0-0_. As per Moss's rule, given that the bandgap of **Cz-BA** is greater than that of **Cz-TBA**, the refractive index of **Cz-BA** is consequently less than that of **Cz-TBA**.

#### 3.4.3 Nonlinear optical parameters

Nonlinearity is a characteristic of a material that manifests its polarizability [64]. As the intensity of an electromagnetic wave incident on the material increases, the material begins to exhibit nonlinear behaviour. As a result, the material's overall polarizability is affected by the electric field in various orders, and the connection between the electric field and polarization vector is articulated as [65].

(12) $$P=\chi^{\left(1\right)}.E+P_{NL}$$

$$\text{or},P=\left[\chi^{\left(1\right)}.E+\chi^{\left(2\right)}.E^{2}+\chi^{\left(3\right)}.E^{3}+\ldots \right]$$

Here χ^(1)^ represents the linear optical susceptibility, and χ^(2)^ and χ^(3)^ denote the second and third-order nonlinear susceptibilities, individually. The inclusion of the first and third-order terms in this expression offers valuable insights into the nonlinear behaviour exhibited by the material.

As per the Miller's empirical rule, the first nonlinear susceptibility (χ^(1)^) and third-order nonlinearity (χ^(3)^) can be computed using the following relations [66]**,**

$$\chi^{\left(1\right)}=\frac{\left(n^{2}-1\right)}{4\pi }$$

(13) $$\text{and}\;\chi^{\left(3\right)}=A\frac{{\left(n^{2}-1\right)}^{4}}{{\left(4\pi \right)}^{4}}=A{\left(\chi^{\left(1\right)}\right)}^{4}$$

Here $n_{0}$ represents the static refractive index at hν → 0 and A = 1.7 × 10^−10^ e.s.u. The computed nonlinear susceptibilities for the material are presented in Table 6.

**Table 6:** Theoretical calculation of refractive index from models for Cz-BA and Cz-TBA samples.

Parameters	*ε*_∞_	χ^(1)^	χ^(3)^	n_2_
**Cz-TB**	5.330	0.344	2.401 × 10^−12^	3.919 × 10^−11^
**Cz-TBA**	5.341	0.345	2.426 × 10^−12^	3.955 × 10^−11^

Nonlinear refractive index ($n_{2}$)

The nonlinear refractive index ($n_{2}$) of **Cz-BA** and **Cz-TBA** samples can be determined utilizing the modified Miller rule proposed by Tichy and Ticha [67]. The empirical relation provides an expression from which the nonlinear refractive index can be deduced, as follows:

(14) $$n_{2}=\frac{12\pi \chi^{\left(3\right)}}{n}$$

The estimated values of n_2_ for **Cz-BA** and **Cz-TBA** is tabulated in Table 6.

## 4 Conclusion

In conclusion, our investigation of carbazole-based dyes, **Cz-BA** and **Cz-TBA**, has unveiled their promising potential for diverse optoelectronic applications, with a particular emphasis on their suitability for DSSC and NLO applications. Through a combination of comprehensive spectral and theoretical analyses, we have shed light on their optical properties, including refractive index calculations and nonlinear optical parameters like the nonlinear refractive index (n_2_), positioning them as promising candidates for nonlinear optical applications. Furthermore, our photophysical studies have underscored their effective light-absorption and emission behaviour, while DFT and TD-DFT simulations have provided robust validation of their electronic characteristics and absorption spectra in congruence with experimental data. The successful design and characterization of these dyes represent a significant leap forward in the practical utilization of advanced organic materials for DSSCs, making substantial contributions to materials science while offering innovative solutions for a wide range of real-world optoelectronic challenges. This marks a significant stride in the practical application of these advanced organic materials in the renewable energy sector.

## CRediT authorship contribution statement

**Praveen Naik:** Writing – original draft, Writing – review & editing, Visualization, Validation, Supervision, Software, Resources, Methodology, Investigation, Formal analysis, Conceptualization. **Nibedita Swain:** Validation, Resources, Methodology, Investigation, Formal analysis, Data curation. **R. Naik:** Resources, Methodology, Investigation, Formal analysis, Data curation. **Nainamalai Devarajan:** Resources, Methodology, Investigation. **Abdel-Basit Al-Odayni:** Resources, Funding acquisition. **Naaser A.Y. Abduh:** Resources. **Kavya S. Keremane:** Writing – review & editing, Validation, Resources. **Devarajan Alagarasan:** Investigation, Formal analysis. **T. Aravinda:** Resources. **H.B. Shivaprasad:** Resources.

## Declaration of competing interest

The authors declare the following financial interests/personal relationships which may be considered as potential competing interests:

Authors declare no conflict of interest If there are other authors, they declare that they have no known competing financial interests or personal relationships that could have appeared to influence the work reported in this paper.
