# Supplementary material for: Exploring optical, electrochemical, thermal, and theoretical aspects of simple carbazole-derived organic dyes
Source: Heliyon. 2024 Feb 6;10(4):e25624. doi: 10.1016/j.heliyon.2024.e25624 (PMC10877267; doi:10.1016/j.heliyon.2024.e25624)
Supplement: Multimedia component 1 [file mmc1.docx]

**Electronic Supplementary Information**

**Exploring Optical, Electrochemical. Thermal, and Theoretical Aspects of Simple Carbazole-Derived Organic Dyes**

**Materials and methods**

All materials and solvents were procured from commercial sources and employed in the reactions without undergoing additional purification steps. Reaction progress was tracked utilizing thin-layer chromatography (TLC), while the molecular structures were definitively confirmed through NMR spectroscopy (Brucker 400 and 100 MHz), HR MS, and elemental analysis (Flash EA1112 CHN analyser). UV-Visible spectra and fluorescence emissions were acquired in tetrahydrofuran (THF) solution at room temperature using a UV-Visible and fluorescence spectrophotometer. In addition, comprehensive Density Functional Theory (DFT) calculations and Molecular Electrostatic Potential (MESP) maps were performed at the B3LYP/6-13 G* level using the BIOVIA Turbomole 2022 software package.

**Spectral Data**

***Figure S1****: ^1^H NMR spectra of* ***Cz-BA***

***Figure S2****: ^1^H NMR spectra of* ***Cz-TBA***


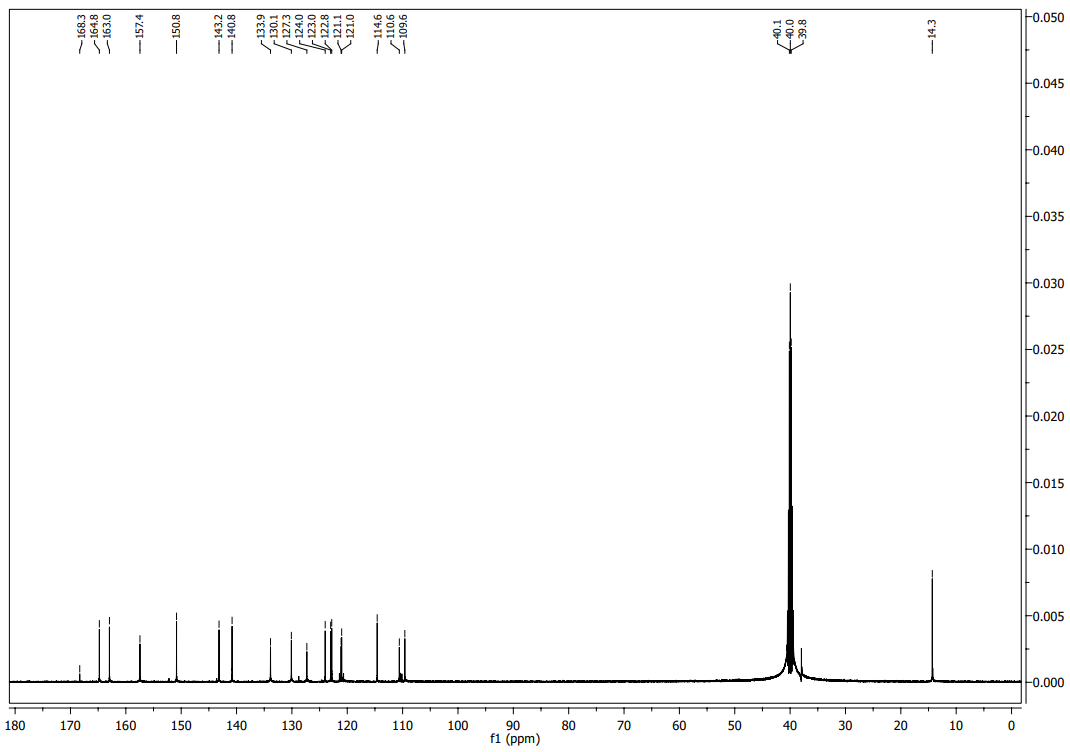


***Figure S3****: ^13^C NMR spectra of* ***Cz-BA***


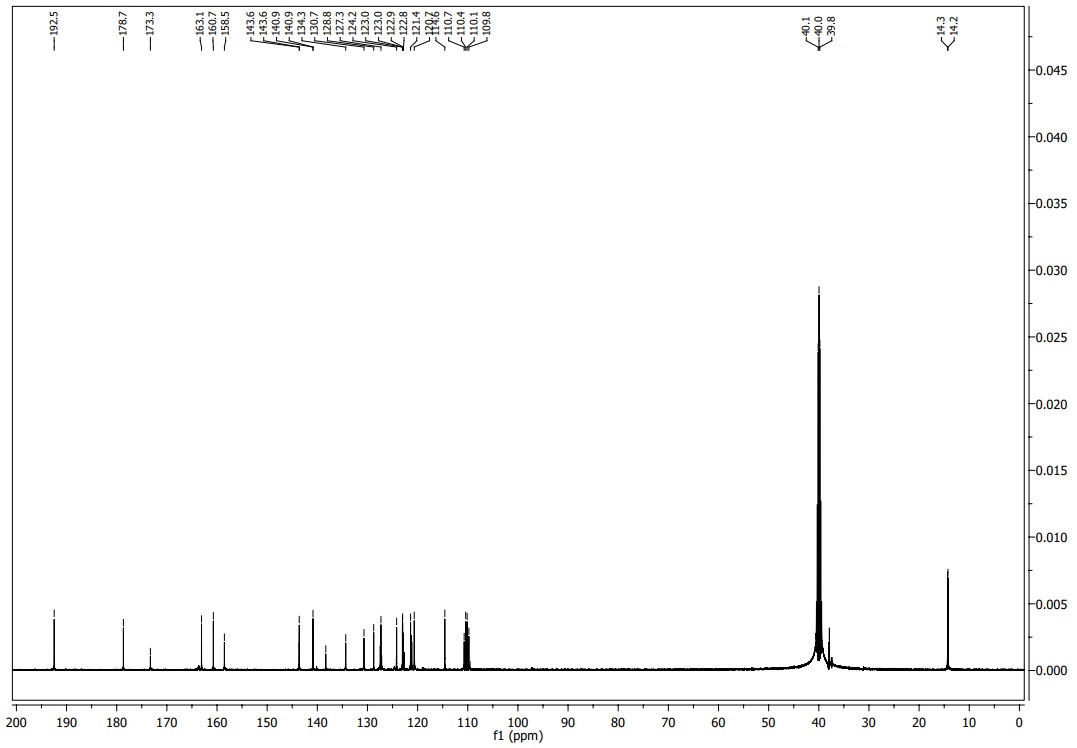


***Figure S4****: ^13^C NMR spectra of* ***Cz-TBA***


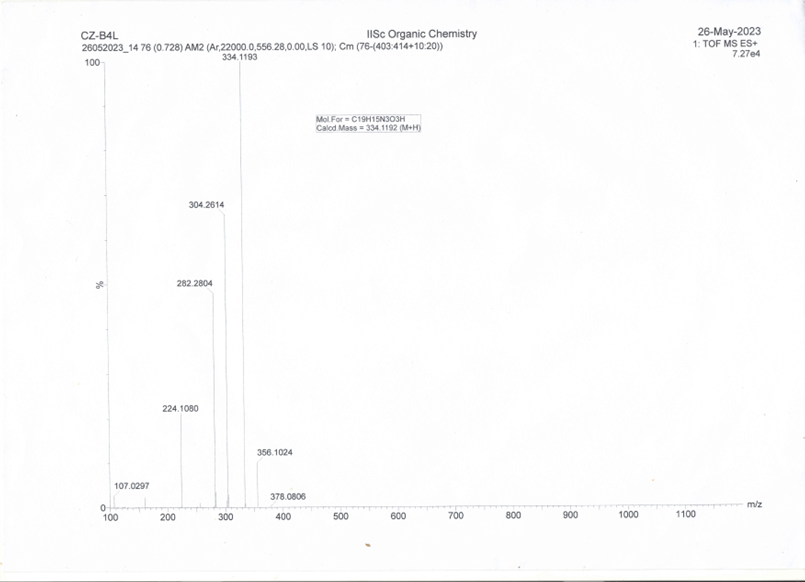


***Figure S5****: HRMS spectra of* ***Cz-BA***


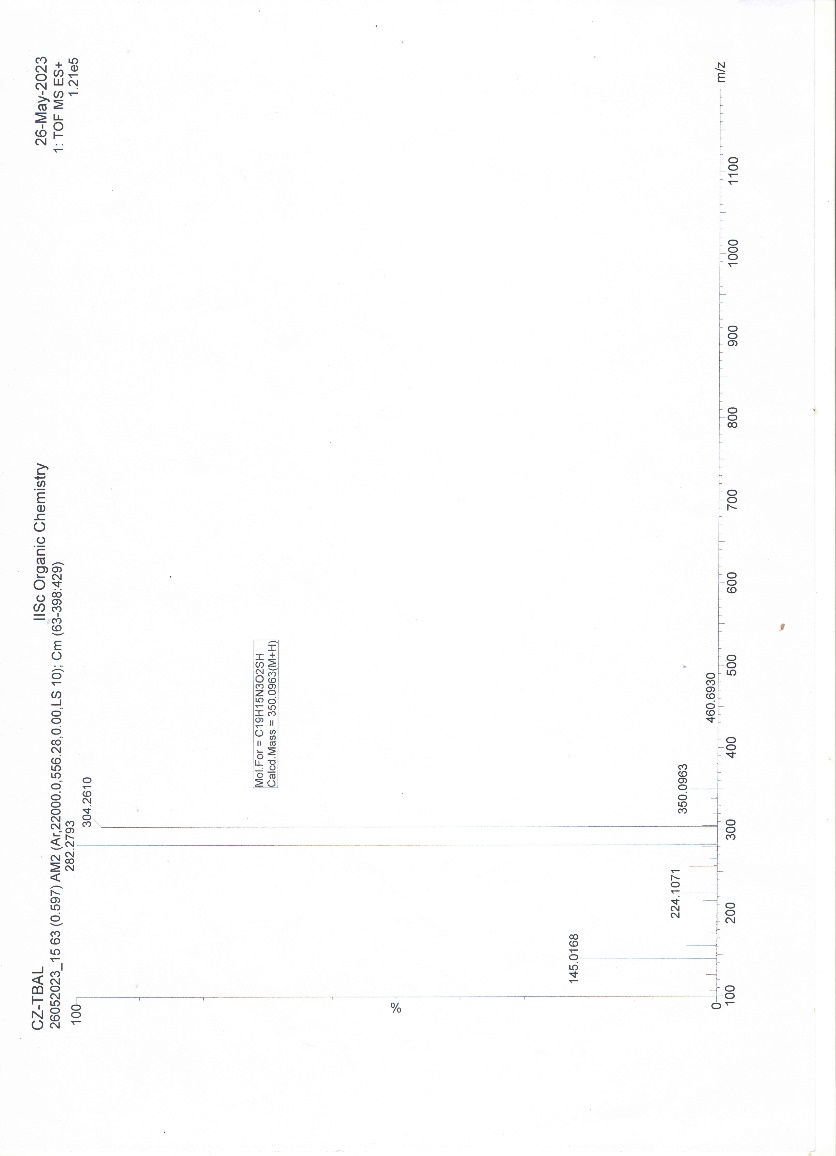


***Figure S6****: HRMS spectra of* ***Cz-TBA***

**CV studies**

**Figure S7:** CV traces of ***Cz-BA*** *and* ***Cz-TBA***

**DFT Calculations**

**Cartesian coordinates of Cz-BA**

ATOM CARTESIAN COORDINATES

1 c -3.00773442277555 1.17225249006995 -0.01509813407110

2 c -0.33662152223346 1.23582730085020 0.39692148670497

3 c 0.38141812185271 3.88444838630061 0.38610511811920

4 c -1.87192022957491 5.27696766725290 -0.02652989544672

5 n -3.89663506203483 3.59240884024546 -0.28378047726262

6 c -4.32426888990326 -1.13422007883711 -0.10525116478905

7 c -2.94255189702027 -3.32763091634830 0.20446637764489

8 c -0.26413483673790 -3.33847226693081 0.61864742569540

9 c 1.01650921403375 -0.98595445517791 0.70782781398934

10 c 2.67842772648962 5.16309916642469 0.67840199961983

11 c 2.69209548951351 7.79684391457828 0.56000103676854

12 c 0.43937454723085 9.14971194352949 0.15409395190671

13 c -1.87397274401525 7.91581713131261 -0.14268591358279

14 c -6.54269998871236 4.32100615456507 -0.63176804668941

15 c -7.91879632213949 4.81545113856880 1.86539874950900

16 c 0.83282040446622 -5.80715204905032 0.90054110693166

17 c 3.18050597082677 -6.87338060619086 1.32901232984960

18 c 5.60285068244333 -5.56462100321287 1.68456021880530

19 n 7.69601794599682 -7.11640171519658 2.10676510685719

20 c 7.77017163180527 -9.71979983760888 2.22539427053147

21 n 5.45347534496622 -10.86475379009430 1.87193610051755

22 c 3.14855264197359 -9.68081699965378 1.43636252617591

23 o 5.95857037104526 -3.26556997646798 1.64913870250597

24 o 1.25067798517501 -10.99726728524221 1.16771041542661

25 o 9.71688847630851 -10.90812397458485 2.60944306359863

26 h -6.35130167589458 -1.21143415236720 -0.40405363631160

27 h -3.92603254300118 -5.12878587502019 0.13807082392568

28 h 3.03541394416540 -0.96331897890758 1.02206690109110

29 h 4.42189485083035 4.12379272351467 0.99384203663476

30 h 4.45916857139635 8.81758295858250 0.78505507792085

31 h 0.49384428830776 11.20084462350227 0.07206224876608

32 h -3.59863666321862 8.98371259322365 -0.44804316245370

33 h -6.57255951805043 6.00315476398688 -1.83282716025640

34 h -7.47245896339966 2.81306286079834 -1.69586074231603

35 h -9.87916178601600 5.36087244891551 1.48377110828569

36 h -7.93389083298014 3.11955460852922 3.05205109263414

37 h -7.01370235613887 6.34561347559260 2.92533452878413

38 h -0.58577112126735 -7.28894658093720 0.73007039436593

39 h 9.36987490842190 -6.21399946398162 2.35494254323389

40 h 5.43138964171169 -12.78067659763592 1.94757816160452

**Cartesian coordinates of Cz-TBA**

ATOM CARTESIAN COORDINATES

1 c -3.01878489067700 1.17403665325573 -0.00956160979894

2 c -0.34593866060727 1.24223659020348 0.40646293166008

3 c 0.36823164638182 3.89237683585948 0.39056094355172

4 c -1.88560734304425 5.27933400255910 -0.03114326932707

5 n -3.90865990314773 3.58872953344923 -0.28568869118108

6 c -4.33479951564174 -1.13503351900665 -0.09450834439710

7 c -2.95301818101066 -3.32463257768007 0.22207459510731

8 c -0.27147082913075 -3.33149363441192 0.63543176037584

9 c 1.00853569717487 -0.97447132753726 0.72242535152047

10 c 2.66251917305984 5.17481912470376 0.68266995488769

11 c 2.67125936278330 7.80843022056435 0.55492703502621

12 c 0.41756271913822 9.15589418381890 0.13936139059098

13 c -1.89344157668756 7.91689035866486 -0.15763334087627

14 c -6.55447574581463 4.31479354436711 -0.64814958725426

15 c -7.93673515241859 4.83070969435066 1.84096401727125

16 c 0.82671155317811 -5.78984945566273 0.91690972818766

17 c 3.19178079538047 -6.84491955810599 1.32866285203229

18 c 5.60260356632470 -5.52986629675339 1.65046467451796

19 n 7.72404170872001 -7.08723380727814 2.04020110192490

20 c 7.79833333311867 -9.65389351464534 2.15132698943356

21 n 5.52208412640866 -10.81102918986351 1.84613675359467

22 c 3.18282583959108 -9.63949146803460 1.44212649542763

23 o 5.97670038223257 -3.23724124087042 1.62020650021264

24 o 1.31043211640500 -10.99255027809727 1.21040944458006

25 s 10.49229353196105 -11.27245725688203 2.62853657770629

26 h -6.36154706219053 -1.21368726464444 -0.39362238149992

27 h -3.93343689016417 -5.12739861324835 0.16052680004973

28 h 3.02729520712584 -0.94576317807795 1.03696902578009

29 h 4.40697463687106 4.13965567431547 1.00527166414877

30 h 4.43604993969993 8.83296723569949 0.77993960838648

31 h 0.46873060256897 11.20669315866453 0.04966404783762

32 h -3.61933673480242 8.98041889816737 -0.47059225403306

33 h -6.57914844331830 5.98661891328915 -1.86353149188001

34 h -7.48065702645742 2.79784227330308 -1.70202811653888

35 h -9.89519819263502 5.37445082749982 1.44786508567058

36 h -7.95724605867175 3.14461570302214 3.04139581881056

37 h -7.03402596543186 6.36897984377714 2.89101702946213

38 h -0.58657561492901 -7.27805022649614 0.76314023066052

39 h 9.38512638958313 -6.15292849304800 2.25927816892066

40 h 5.48970112978593 -12.72753730412368 1.91829408071186
